# TEM sample preparation using micro-manipulator for in-situ MEMS experiment

**DOI:** 10.1186/s42649-021-00057-8

**Published:** 2021-06-09

**Authors:** Hyunjong Lee, Odongo Francis Ngome Okello, Gi-Yeop Kim, Kyung Song, Si-Young Choi

**Affiliations:** 1 Korea Institute of Industrial Technology, Incheon, 21999 South Korea; 2 Department of Materials Science and Engineering, Pohang University of Science and Technology (POSTECH), Pohang, 37673 South Korea; 3 Division of Materials Testing and Reliability, Korea Institute of Materials Science, Changwon, 51508 South Korea

**Keywords:** Manipulator, MEMS, In-situ transmission electron microscopy

## Abstract

Growing demands for comprehending complicated nano-scale phenomena in atomic resolution has attracted in-situ transmission electron microscopy (TEM) techniques for understanding their dynamics. However, simple to safe TEM sample preparation for in-situ observation has been limited. Here, we suggested the optical microscopy based micro-manipulating system for transferring TEM samples. By adopting our manipulator system, several types of samples from nano-wires to plate-like thin samples were transferred on micro-electro mechanical systems (MEMS) chip in a single step. Furthermore, the control of electrostatic force between the sample and the probe tip is found to be a key role in transferring process.

## Introduction

In the primary stage of TEM observation, utilizing TEM for material characterization was confined to static structure observation at ambient conditions because of the technical limitations such as high-level vacuum system. However, according to the rising interests on nanomaterials and resulting requirement for simultaneous understanding of nano-scale reaction or phenomena with material properties, in-situ TEM techniques has been proposed for understanding dynamics. Accordingly, special types of TEM holders using MEMS-based system were developed and that enabled application of external stimulus on TEM samples such as heating, biasing, or mechanical stress even at 10^− 5^ ~ 10^− 7^ Pa vacuum condition (Novák et al. 2016; Tochigi et al. 2019; Li et al. 2020; Mele et al. 2016). Therefore, real time observation of instantaneous microstructure evolution such as phase transition, electrical and magnetic domain switching, electrochemical reactions, and other incredible changes became possible (Novák et al. 2016; Tochigi et al. 2019; Mele et al. 2016). Especially, MEMS contain multiple necessary sensors and actuators for manipulating stimuli in one platform making it an appropriate system for TEM, which has a small pole-piece gap. Furthermore, MEMS apply stimulus in micrometer scale small area that fine and fast control of stimulus could be achieved (Roshan et al. 2016; Damiano et al. 2008).

Despite these merits, TEM sample preparation for in-situ observation leads to the great challenge resulting from the small dimension of a few micron-scaled MEMS window transmittable in TEM. Therefore, the samples have been tried to be precisely transferred on the window of MEMS in many ways. Typically, FIB (Focused ion beam) technique or drop casting method are representative tools for transferring samples on MEMS windows (Vijayan et al. 2017; Vijayan 2018). On one hand, FIB sampling is useful because of relatively easy handling and site-specific preparation of sample. However, FIB technique inevitably accompanies Ga^+^ ion-induced damage on the MEMS despite the short observation. Especially when MEMS chips applied to in-situ gas or liquid experiments, the damaged MEMS chips can cause the critical failure during in-situ observation. In addition, Ga^+^ ion implantation could lead to several problems including leakage current, structure destruction, or interruption of atomic scale imaging in TEM (van Omme et al. 2018; Mayer et al. 2007; Prenitzer et al. 2003). In drop casting method, although nano-scaled materials are transferred on MEMS window without any ion-induced damage, but the accurate control of sample on window is limited and additional contamination such as agglomeration could be problematic (Vijayan et al. 2017; Unocic et al. 2010).

Here, we suggested the optical microscopy based manipulating system for in-situ TEM using MEMS chips. By controlling the electrostatic force between tungsten manipulating tip, sample, and substrate, the precise transfer of different sample geometries including micro-sized 2D flake, nanowire, and FIB lamellar have been demonstrated. Transferring process is observed by real-time optical microscopy and transferred samples are confirmed by TEM observation and corresponding the selected area electron diffraction (SAED) patterns.

## Materials & methods

**Fig. 1:**
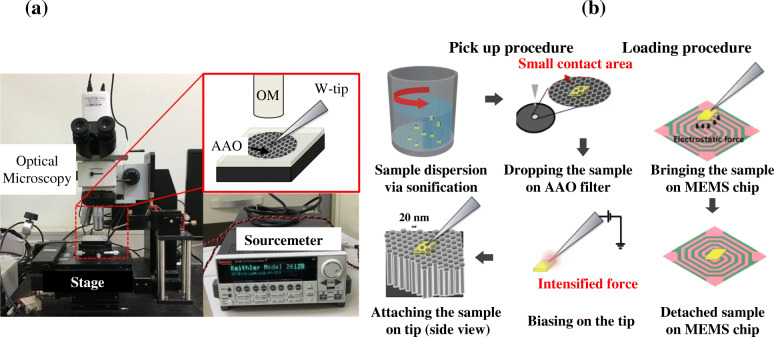
**a** Schematic configurations of manipulating system composed of optical microscopy, stage, and sourcemeter. Magnified red-squared region indicates the AAO of 20 nm pores that is put below the sample to reduce the contact area of sample and substrate. **b** Schematic illustrations of transferring procedure which is divided into pick-up and loading process

Figure 1 shows the transfer method of TEM samples using our micro-manipulating system. First of all, manipulating system is comprised of optical microscopy, stage, and interchangeable tungsten manipulating tip (GGB INDUSTRIES, INC., FLORIDA, USA) as shown in Fig. 1A. Also, the system includes a voltage sourcemeter (Model 2612B, KEITHLEY, Cleveland, USA), which is directly connected to tungsten tip for tip biasing. Anodic aluminum oxide (AAO) membrane filter with a pore diameter of 20 nm (WhatmanTM, Connecticut, USA) is put on the stage to be used as a substrate in pick-up procedure. This manipulating system could be applied on the other types of MEMS chip. In this work, we use a MEMS heating chip (DENS solutions, Delft, Netherlands) (Damiano et al. 2008) for the demonstration, the target of sample to be transferred on, for TEM observations. Figure 1B illustrates overall transferring procedure of TEM sample that is separated into pick up and loading procedure. In the pick-up procedure, Fig. 1B illustrates overall transferring procedure of TEM sample that is further separated into pick up and loading procedure.

Initially in the pick-up procedure, (i) the sample to be transferred, which is h-BN 2D flakes or KNbO_3_ nanowires, is dispersed in ethanol via 10 min sonicating and dropped on AAO filter. Because of the honeycomb-like structure of AAO with a diameter of 20 nm, the contact area between the sample and substrate is minimized thus facilitating the attachment of the sample to the tip. Next, (ii) select the appropriate size of tungsten tip i.e., similar with sample size (tip radius of 100 nm), to cause proper amounts of electrostatic force. Small size of tip compared to sample cannot attach the sample, but large size one cannot detach the sample by strong electrostatic force. Generally, samples are attached on tip in this procedure by naturally generated electrostatic force between tip and sample. (iii) When the sample is not attached on tip, we can enhance the electrostatic force between tip and sample through 0.1v to 1v biasing on tip. In loading procedure, to detach the sample on target area, (iv) we carefully move the sample to the target area. Then larger electrostatic force between sample and target area in MEMS chip than that of sample and tungsten tip, which is attributed to larger size loading substrate than tip, detaches the sample.

In the case of FIB transferring process, we omit the procedure of (i) and simply attach the free standing sample from grid by using the larger size of tip (radius of ~ 1000 nm). To put the FIB sample on the tip easily, side carbon deposition part of sample was almost eliminated by 30 kV accelerated Ga^+^ ion beam at beam current of few pA (JIB-4601F, JEOL, JAPAN). Whole transferring process is observed by optical microscopy and transferred samples are confirmed by 200 kV accelerated TEM (JEM-2100, JEOL, JAPAN) image and SAED patterns.

Although the electrostatic force generated at room temperature is strong enough to bond the samples on to MEMS chip, it should be noted that for heating or electrical biasing, after transferring the nanowire and FIB sample on to the MEMS chip, the metallic contact should be reinforced via FIB or lithography to avoid huge sample drift during the experiment.

Our sample transfer method exhibits some similarities with the work previously reported by Lucille et al. (Giannuzzi et al. 2015), however, we attempted to investigate the electrostatic attraction between the probe and sample for precise operation of the manipulator.

## Results & discussion

**Fig. 2:**
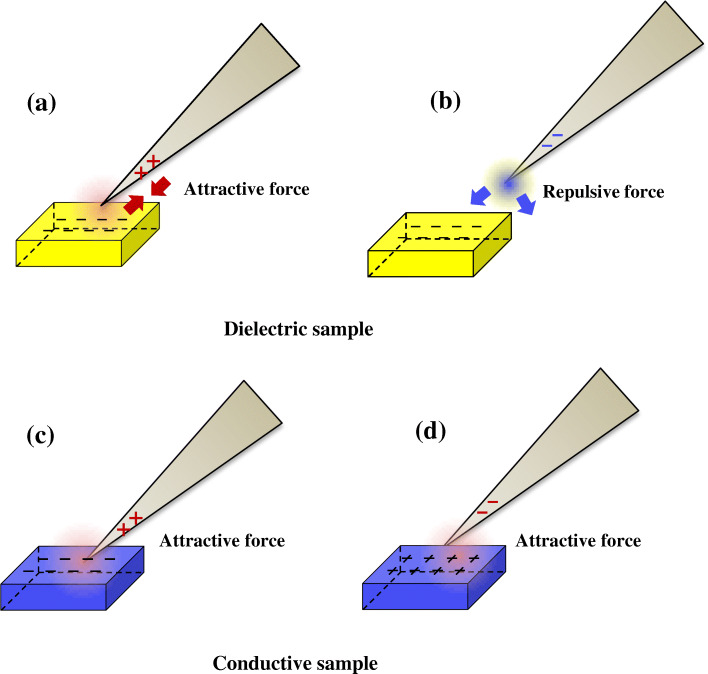
Schematic illustrations of mechanism of intensifying electrostatic force via biasing on tip (**a**-**b**) **a** attractive force induced by positive charge biasing and **b** repulsive force by negative charge biasing on tip where the surface of dielectric sample has negative charges. **c**-**d** Attractive force in conductive specimen induced by both **c** positive charge biasing and **d** negative charge biasing on tip

Figure 2 schematically illustrates the principle of intensifying electrostatic force between sample and tip which is divided into two cases depending on the dielectric or conductive nature of the sample. In case of dielectric sample, charges of signs are previously decided on sample surface as attractive or repulsive force which could be controlled by tip biasing. If sample surface is negatively charged, the tip should be biased by a positive voltage to make attractive force (Fig. 2A). Otherwise if the negative bias is applied to tip, a repulsive force inhibits the attaching process because of same sign of charge (Fig. 2B). Thus, it is suggested that finding proper sign of biasing to attach the sample in dielectric case must be considered. On the other hand, the sign of charges on the conducting surfaces can be easily changed different from dielectric one. Irrespective of applied sign of charges in tip biasing, surface charge of sign in conducting sample is easily flipped off from easy mobility of electron. In other words, attaching procedure in a conductive sample is only decided by applied biasing voltage on tip (Fig. 2C, D).

**Fig. 3:**
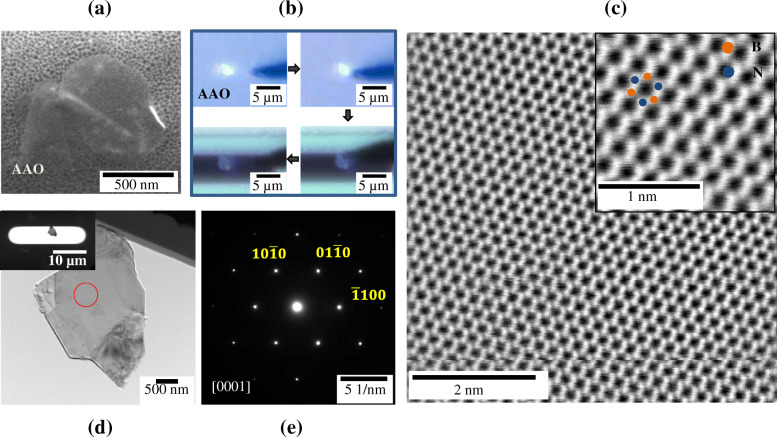
Transferring process of h-BN 2D flakes on MEMS window from AAO filter **a** SEM image of h-BN flakes dispersed on AAO. **b** Transferring process observed by optical microscopy in real time using tip of radius < 100 nm. **c** TEM image of transferred h-BN single flake on MEMS window. The inset shows low magnification TEM image of sample. **d** Corresponding SAED pattern of red circled part in **c**. **e** STEM-BF image of transferred h-BN flake. Inset reveals the high-magnification image of atomic column of B and N

Primarily, the micro-sized h-BN flake is transferred on MEMS window using the method described in (Fig. 3). The SEM image of Fig. 3A shows a well-dispersed h-BN flake on AAO filter. The overall transferring process of h-BN flake is demonstrated by optical microscopy (Fig. 3B). It is evident that a few microns of h-BN single flake can be easily and successfully transferred on window via our suggested method. Figure 3C and D show the low magnification TEM images of single h-BN flake and its SAED pattern. Although the h-BN flake is very thin as electron transparent, it is successfully transferred on MEMS window without any mechanical damage. SAED pattern of h-BN obtained clearly indicates single-crystalline hexagonal array that implicates an accurate transferring process of single flake as shown in Fig. 3D. High-resolution STEM (Scanning Transmission Electron Microscopy) image of h-BN flake presents clear atomic columns of boron and nitrogen. In other words, the proposed manipulating system could enable damage-free selective transfer of a single flake that is sufficiently thin enough for atomic scale observation (Fig. 3E). The transferred flake is well adhered on window in place during TEM observation regardless of 200 kV-accelerated high energy of electron beam.

**Fig. 4:**
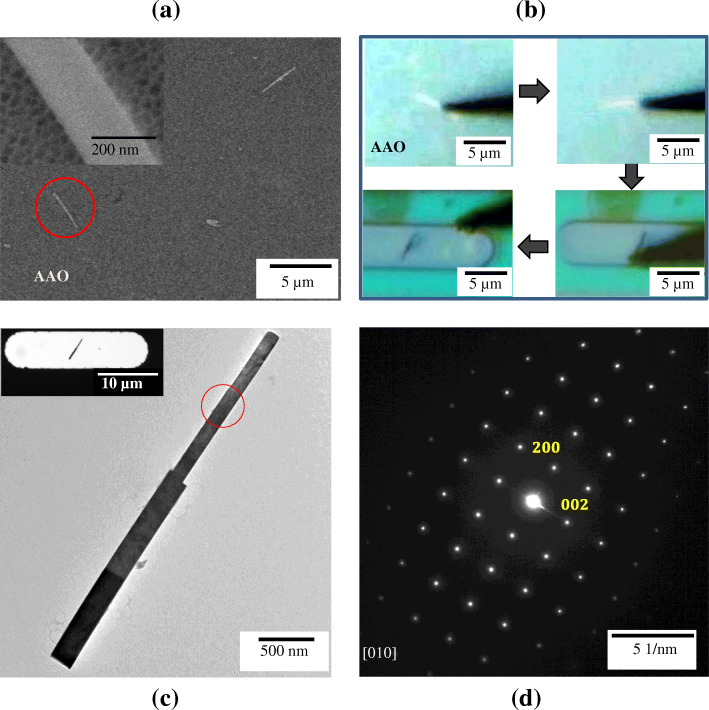
Transferring process of KNbO_3_ nanowire on MEMS window from AAO filter **a** SEM image of KNbO_3_ nanowires dispersed on AAO. The inset shows 20 nm pore arrays of AAO. **b** KNbO_3_ nanowire transferring process observed by optical microscopy in real time using tip of radius < 100 nm. **c** TEM image of transferred KNbO_3_ single nanowire on MEMS window. The inset shows low magnification TEM image of sample. **d** SAED pattern of red circled part in **c**

Figure 4A shows SEM image of well dispersed KNbO_3_ nanowires on AAO filter. The average length and diameter of KNbO_3_ nanowires are 5 μm and 200 nm, respectively, and contact area and electrostatic force between AAO template and KNbO_3_ nanowire could be effectively minimized (inset of Fig. 4A). Subsequently, the KNbO_3_ nanowire was transferred to MEMS chip, which was directly observed as a bright line by optical microscopy as shown in Fig. 4A and B. Likewise to the case of h-BN flake, KNbO_3_ nanowire on AAO filter is attached on tungsten tip and transferred on SiN_x_ window in simple one-step. As a result of TEM observation, the KNbO_3_ was accurately fixed on transparent window of MEMS chip and successfully transferred without damages such as mechanical breakage and bending, despite its nanometer-sized diameter (inset of Fig. 4C). Figure 4D shows SAED pattern of transferred nanowire from Fig. 4C, which is the single-crystalline.

**Fig. 5:**
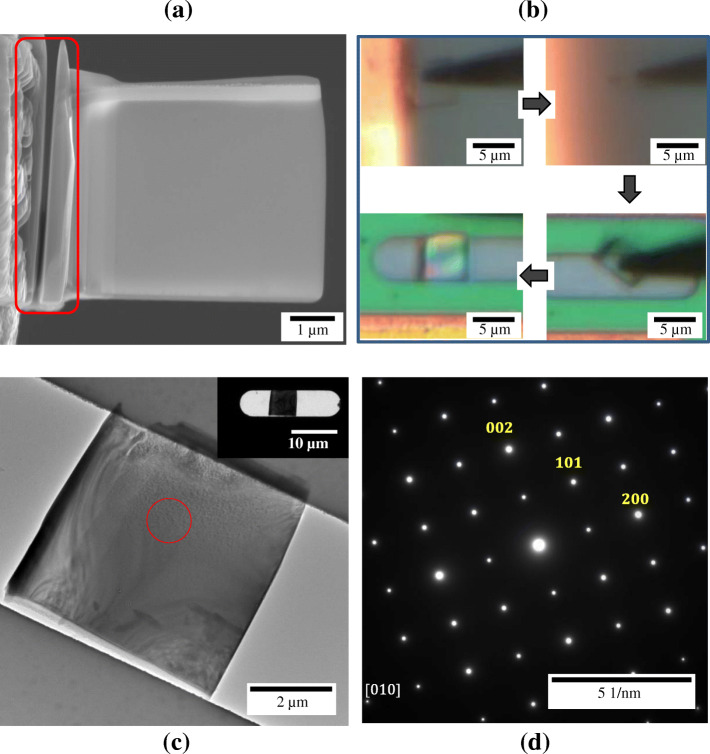
Transferring process of YAlO_3_ pre-milled FIB sample on MEMS window from Cu grid **a** 53 degree tilted SEM image of YAlO_3_ FIB sample. Red squared part shows cut FIB side by 30 kV accelerated Ga^+^ ion. **b** Transferring process observed by optical microscopy in real time using tip of radius < 1000 nm. **c** TEM image of transferred YAlO_3_ FIB sample on MEMS window. The inset shows low magnification TEM image of sample. **d** SAED pattern of red circled part in **c**

Moreover, we also transferred a lamellar type YAlO_3_ sample prepared by conventional FIB procedure to MEMS chip from Cu grid as shown in Fig. 5. Although the TEM sample could be directly prepared on MEMS chip by using FIB for in-situ TEM experiments, FIB milling inevitably accompany re-deposition of sample or Ga^+^ ion penetration on MEMS chip especially during final milling (Vijayan et al. 2017). Particularly, in the cases of the gas-or liquid-environmental holders the final milling can cause serious damage to the sealing membrane and thus in-situ experiments are no longer available with the damaged membrane (Roshan et al. 2016). Therefore, to prevent or minimize ion-induced contamination or damage in MEMS-based in-situ TEM experiments, we suggest that lamella type samples be prepared by FIB technique on Cu grid and transferred to MEMS window using micro-manipulation system. In order to attach the FIB-milled sample on manipulating tip from Cu-grid, we cut the side of FIB sample using pA Ga^+^ ion source as shown in Fig. 5A. In Fig. 5B, the whole transfer process after side milling is shown by optical microscopy. In this case, larger size of tip compared to the above cases was used to attach the free standing FIB sample due to the large dimension of FIB sample. Figure 5C shows TEM image of the transferred YAlO_3_ FIB and the inset shows the low magnification image of sample on window. Corresponding SAED pattern in Fig. 5D shows YAlO_3_ FIB sample is sufficiently thin for TEM observation common with the other samples. In this case, it appears to have little bend-contour in TEM sample, which could have occurred during tip attachment process owing to the large electrostatic force. Therefore, it is suggested that use of appropriate tip size and biasing voltage is important, considering FIB sample dimension in each case.

## Conclusions

We have proposed the optical microscopy-based micro- and nano-sized materials transferring system for in-situ TEM observations. By applying the bias to manipulating tip, we successfully transferred to MEMS chip in a one-step procedure TEM sample of different geometries, such as 2D flake, nanowire, and lamella sample prepared by FIB technique. In this method, we can prepare TEM sample for in-situ observations very quickly and easily without FIB works and any damage or contamination. Also, we demonstrated that transferred samples were electron transparent but less damaged, enabling the atomic-level STEM imaging. We believe that our results will provide the useful and simple TEM sample preparation methods for in-situ TEM observations.

## Data Availability

Please contact author for data requests.
